# The effects of extracorporeal shock wave therapy vs hand massage on serum lipids in overweight and obese women

**DOI:** 10.1016/j.amsu.2021.01.005

**Published:** 2021-01-15

**Authors:** Kyung.Jin Lee, Jin.Ik Park, Soo.Yeon Oh

**Affiliations:** a Department of Beauty Art, Youngsan University, Busan, Republic of Korea; b Department of Dermatology, Yangsan Kangnam Clinic, Yangsan, Republic of Korea

**Keywords:** Obesity, Overweight, Extracorporeal shock wave therapy, Body composition, Cholesterol, Abdominal obesity

## Abstract

**Background:**

The purpose of our study was to investigate the effects of extracorporeal shock wave therapy (ESWT) and hand massage therapy (HMT) on serum lipids and body composition in Korean women.

**Materials and methods:**

We randomly classified 60 participants into overweight and obese groups. Subjects received ESWT and HMT twice a week for six weeks (a total of 12 sessions).

**Results:**

Body weight and body mass index decreased significantly in obese women from both groups. Waist circumference significantly declined in obese women and overweight women in both treatment groups (p < 0.001). Body fat significantly decreased in the ESWT group of obese women (p < 0.01), while a significant reduction in abdominal obesity was noted only in the HMT group of overweight women (p < 0.01) and the ESWT group of obese women (p < 0.01). There was a significant decrease in triglycerides in the ESWT group of obese women (p < 0.01).

**Conclusions:**

These results suggest that ESWT and HMT could be helpful for the management of people with excess abdominal fat and obesity. Moreover, ESWT is more effective than HMT for improving abdominal obesity and triglyceride levels in obese women as compared to overweight women.

## Highlights

• ESWT has the effect of reducing triglycerides in obese people.
• ESWT on weight loss were more pronounced in obese participants than overweight.
• The reduction of BMI in ESWT was more pronounced in obese patients than overweight.

## 1 Background

As abdominal obesity and dyslipidemia are key factors driving the increased prevalence of metabolic syndrome in Korea over the last ten years, lifestyle changes should be promoted at the national level in order to decrease the burden of and issues related to the metabolic syndrome [1]. In the 2003–2004 National Health and Nutrition Examination Survey (NHANES), 28.5% of adults between ages 20 and 39 were obese, compared with 36.8% of adults ages 40 to 59, and 31.0% of those older than 60 [2]. The increasing prevalence of overweight status and obesity will in turn lead to gradual increases in disease prevalence [3]. A World Health Organization (WHO) Expert Consultation concluded that the proportion of Asians with a high risk of type 2 diabetes and cardiovascular disease (CVD) is considerable at a body mass index (BMI) lower than the WHO's existing cutoff for the definition of overweight (>25 kg/m^2^) [4]. Therefore, it is imperative to recognize and examine overweight people, as overweight status can lead to obesity. Given that all-cause mortality is significantly higher in obese people (BMI ≥ 30) than in people with a normal weight, and being overweight (BMI of 25 to < 30) is associated with a significant decrease in all-cause mortality [5], weight loss from obesity to overweight status alone markedly lowers the prevalence of diseases caused by obesity.

In the Korean Heart Study (KHS), dyslipidemia was one of the most common risk factors for CVD in women, along with hypertension, diabetes, and smoking [6]. Approximately 17 million people worldwide die each year from CVD, representing 30% of all fatalities [7]. Given that obesity aggravates the majority of risk factors for CVD, it is not surprising that the rates of most CVDs, including coronary heart disease, hypertension, atrial fibrillation, and heart failure, are higher in obese people [8]. Many studies have validated the correlation between abdominal fat and metabolic dysfunction, and waist circumference has become a standard for metabolic syndrome diagnosis [9]. Weight loss is beneficial for improving serum lipids in overweight and obese people [10], and, in particular, a reduction in abdominal fat may lower the risks of dyslipidemia and chronic diseases [11,12]. As many studies have indicated a reduction in total cholesterol, low-density lipoprotein cholesterol (LDL-C), and triglyceride levels in the blood during weight loss in obese and overweight people [10], efforts to improve obesity is crucial from the perspective of CVD prevention. The management of abdominal obesity involves different techniques, such as pharmacologic treatments, exercise and diet, behavioral change, surgery, devices, and massage [13,14].

Extracorporeal shock wave therapy (ESWT) has been used as a treatment for kidney stones since 1980 [15]. Next, it was proven effective in treating orthopedic conditions [16], and it is now also widely used to treat cellulite [17,18]. Shock wave therapy employs the physical forces of shock waves to stimulate cavitation without damaging the skin or organs [19]. Shock waves generated outside the body are a noninvasive means of delivering therapeutic energy to a restricted area of the body [17]. ESWT improves cellulite [17] by enhancing cell scaffolds, especially in dermal and subcutaneous fat tissues, instead of by reducing subcutaneous fat.

Hand massage therapy (HMT) generally improves the long-term recovery of muscle damage from intensive exercise, accelerating healing by enhancing blood flow in the strengthened muscles [20], and it is an efficient alternative approach for patients who have high blood pressure due to stress [21].

ESMT and HMT are commonly used for abdominal fat control in Korean clinics, but no studies have examined their effects according to overweight status and obesity. The current study analyzes the impacts of ESWT and HMT on body composition and serum lipids separately in obese and overweight women.

## 2 Materials and Methods

### 2.1 Research subjects and period

The inclusion criteria were a BMI of 23 kg/m^2^or higher, a waist-hip ratio (WHR) of 0.80 or greater, and a waist size of 80 cm or longer. In order to recruit to the study patients who were hospitalized or visiting K Hospital, in-hospital promotion was conducted for those who met the participation requirements in prescreening. Only the patients who read the clinical trial data sheet and gave written authorization for participating in the study were included. Participants were notified that shock wave treatment and hand massage therapy procedures could have bruising or other side effects. All significant harm or unintended effects of each group were reported to the Institutional Review Board (YSUIRB-201510-BR-003-02). The work has been reported in line with Consolidated Standards of Reporting Trials (CONSORT) Guidelines. And this paper was conducted by applying Research Registration (Unique Identifying Number: KCT0004999, https://apps.who.int/trialsearch/).

**Fig. 1:**
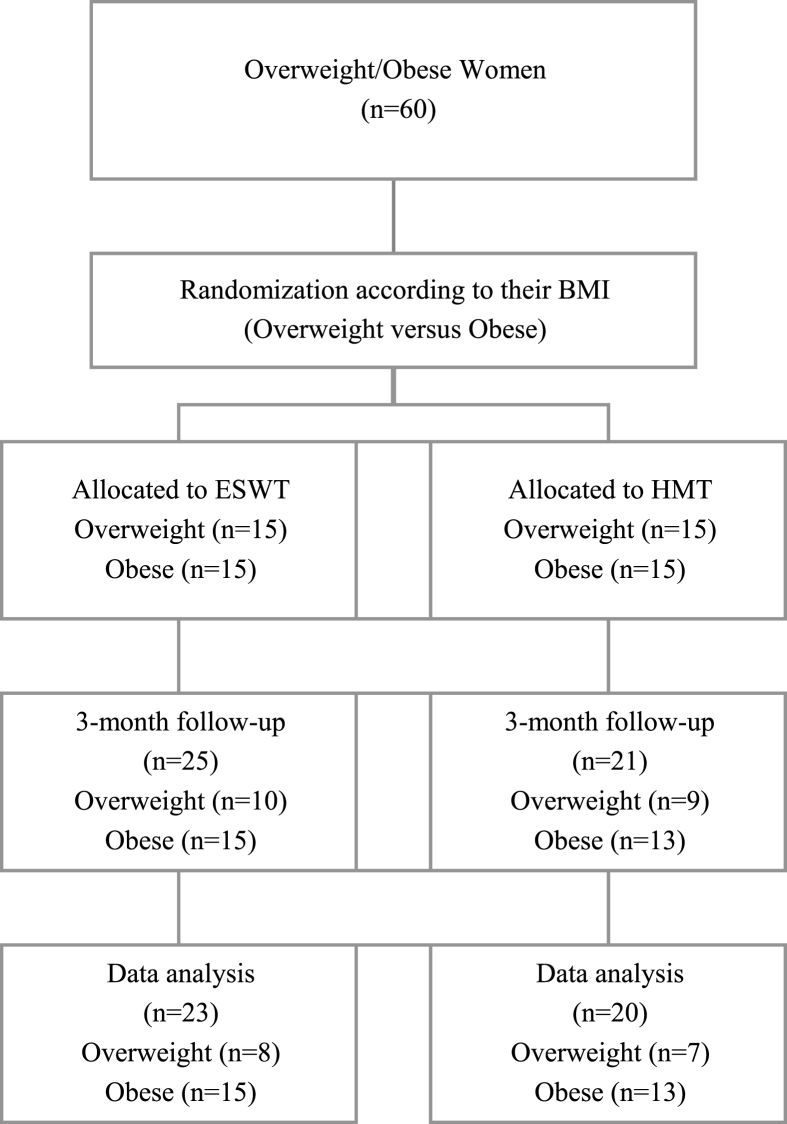
Study flow diagram ESWT; extracorporeal shockwave therapy, HMT; hand massage therapy.

The sample size was calculated based on G*Power 3.1.3. Participants were asked to pick a piece of paper, and then they were randomly assigned to the ESWT group (*n* = 30) if their paper read “O” or to the HMT group (*n* = 30) if their paper read “X.” During the course of the research study, five participants from the ESWT group and nine participants from the HMT group were excluded because of personal reasons. Therefore, a total of 46 participants finished all 12 sessions, which were conducted two times per week for six weeks, between Feb. 1 and May 31, 2016. The final analysis included data from 43 patients, excluding three with abnormally high serum lipid levels (Fig. 1).

## 3 Methods

### 3.1 Procedure

#### 3.1.1 Treatment with extracorporeal shock wave

Shock wave treatment was carried out using a Joeun Medical ESWT (Model No. JESL-1000, Joeun Medical, Korea, 2013) with a head unit of Ø40 mm, 5-bar shock pressure, 10-Hz wave frequency, and 4000 shocks. Each therapy session lasted 20 min, including treatment preparation (the ESWT was turned on, and the head sterilized), the application of cream to the participant's abdomen in order to decrease surface friction and ensure smooth rubbing, speed adjustment by pressing the foot switch, the rolling of the head over the abdomen, and the administration of a medical hot pack.

#### 3.1.2 Hand massage therapy

HMT was conducted by three professional, nationally certified hand therapists, with each session lasting 20 min. The therapists applied massage cream to the participant's bare abdomen, with a breathing step following, and then used five manual techniques (stroking, rubbing, kneading, tapping, and shaking), finishing with a hot pack.

### 3.2 Instruments

#### 3.2.1 Subjective measure

Participants were asked to complete a diet and exercise log to demonstrate maintenance of their usual diet and exercise regimen before participating in the study; this step was taken in order to avoid the confounding effects of diet and exercise by controlling for external variables.

#### 3.2.2 Physiological measurements

A study nurse used a Genius 220 PLUS (Jawon Medical, Korea, 2010) to measure body composition, including weight (kg), BMI (kg/m^2^), body fat proportion (percent), and WHR. The nurse measured waist size at the body midsection (iliac crest), which is between the lowest point of the ribs and the highest point of the pelvis, while the participants were in a calm, exhaling position, with their legs 25 cm–30 cm apart. Measurements were recorded in millimeters using a Hoechstmass tape measure (made in Germany). The study nurse also took blood samples from each participant twice—one before the first treatment, and the other following the last treatment—after the participant fasted for 12 h at K Hospital. Measurements included triglycerides, total cholesterol (TC), LDL-C, and high-density lipoprotein cholesterol (HDL-C), which were taken using the Hitachi 7600-110 (Japan, 2014), an automated biochemistry analyzer, and cortisol, which was measured using the Roche Cobas 8000 (Switzerland 2014), an immunology analyzer.

## 4 Data analysis

Data were analyzed using version 23.0 of the Statistical Package for the Social Sciences (SPSS) for Windows, and the results are given as mean ± standard deviation (SD). Shapiro–Wilk testing was conducted to evaluate normality before comparing the impact of ESWT and HMT based on obesity level. The pretest-posttest difference was analyzed using the paired *t*-test, and the independent *t*-test was used to assess the distinctions in posttreatment effects between the two groups.

## 5 Results

### 5.1 Body measurements

The mean ages of the ESWT and HMT groups were 43.7 ± 5.7 years and 44.5 ± 4.9 years (range 36–53 years), respectively. The mean height of the study participants was 162.5 cm, and the mean weight was 70.6 kg.

**Fig. 2:**
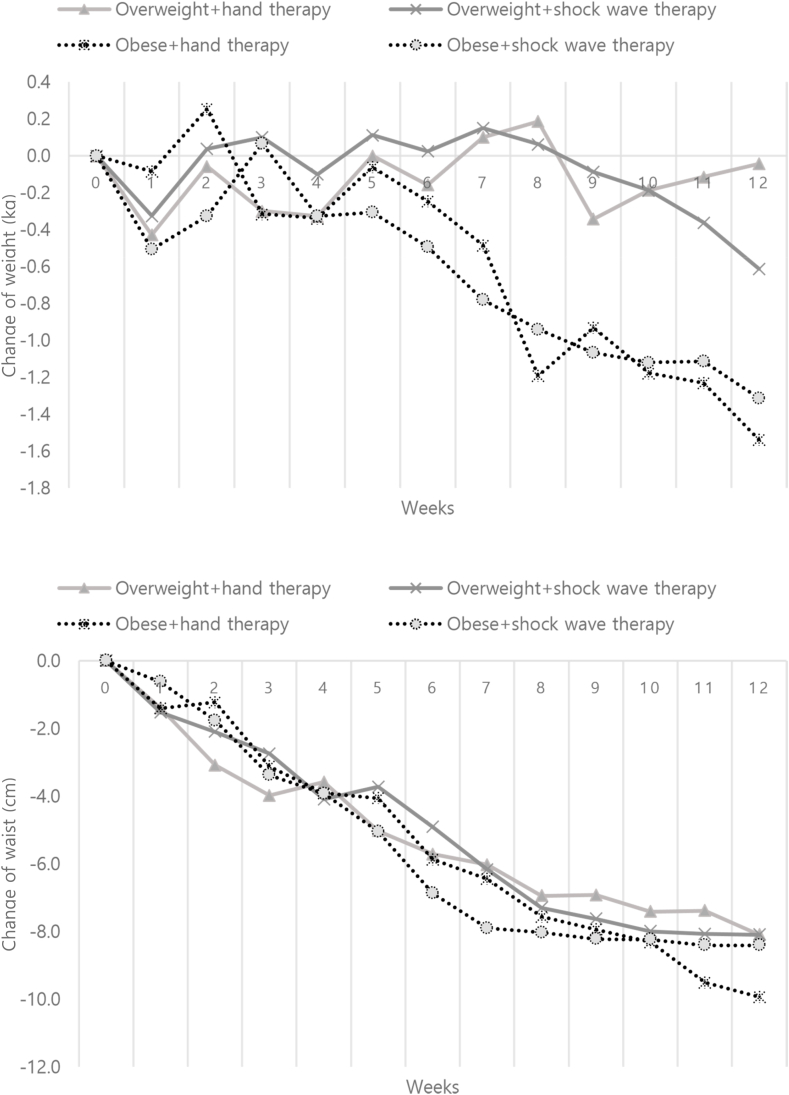
Changes in weekly body weight and waist of the participants.

Table 1 shows changes in participants' body composition during the study. The effects of the six-week (12-session) ESWT and HMT programs on weight loss were more pronounced in obese participants than in overweight participants. Body weight decreased considerably—from 74.6 ± 2.6 kg to 73.0 ± 2.3 kg among obese patients in the HMT group (*p* < 0.01), and from 74.2 ± 2.4 kg to 72.9 ± 2.3 kg among obese patients in the ESWT group (*p* < 0.01). Obese participants from both the ESWT and the HMT groups showed sustained weight loss following the fifth session, and both overweight and obese participants from the two intervention groups exhibited sustained reduction in waist circumference (Fig. 2).

**Table 1:** Body compositions changes in participants during the trial.

		Baseline			12 weeks			Difference
Body weight	Overweight + HMT (n = 7)	62.0	±	1.3	61.9	±	1.4	−0.04
	Overweight + ESWT (n = 8)	65.2	±	1.6	64.6	±	1.5	−0.61
	Obese + HMT (n = 13)	74.6	±	2.6	73.0	±	2.3**	−1.54
	Obese + ESWT (n = 15)	74.2	±	2.4	72.9	±	2.3**	−1.31
BMI	Overweight + HMT (n = 7)	23.8	±	0.2	23.8	±	0.3	−0.05
	Overweight + ESWT (n = 8)	24.0	±	0.3	23.7	±	0.3	−0.24
	Obese + HMT (n = 13)	28.1	±	0.8	27.5	±	0.8*	−0.55
	Obese + ESWT (n = 15)	28.3	±	0.6	27.8	±	0.6**	−0.57
Percent of body fat	Overweight + HMT (n = 7)	30.5	±	0.7	30.2	±	1.2	−0.26
	Overweight + ESWT (n = 8)	31.7	±	0.9	31.5	±	0.6	−0.19
	Obese + HMT (n = 13)	36.2	±	0.8	35.6	±	0.9	−0.53
	Obese + ESWT (n = 15)	36.3	±	0.7	35.7	±	0.6**	−0.65
Waist	Overweight + HMT (n = 7)	86.1	±	1.4	78.0	±	1.7***	−8.10
	Overweight + ESWT (n = 8)	90.3	±	1.7	82.2	±	1.7***	−8.10
	Obese + HMT (n = 13)	97.3	±	1.5	87.4	±	2.2***	−9.94
	Obese + ESWT (n = 15)	95.6	±	1.4	87.2	±	1.3***	−8.41
Abdominal obesity	Overweight + HMT (n = 7)	0.83	±	0.01	0.81	±	0.01**	−0.02
	Overweight + ESWT (n = 8)	0.82	±	0.01	0.82	±	0.00	0.00
	Obese + HMT (n = 13)	0.87	±	0.01	0.86	±	0.01	−0.01
	Obese + ESWT (n = 15)	0.87	±	0.01	0.86	±	0.01**	−0.01

*p < 0.05, **p < 0.01, ***p < 0.001 compared with baseline.

ESWT; extracorporeal shockwave therapy, HMT; hand massage therapy.

The waist circumference of overweigh participants significantly decreased from 86.1 ± 1.4 mm to 78.0 ± 1.7 mm in the ESWT group (*p* < 0.001), and from 90.3 ± 1.7 mm to 82.2 ± 1.7 mm in the HMT group (*p* < 0.001). In obese participants, waist circumference significantly decreased from 97.3 ± 1.5 mm to 87.4 ± 2.2 mm in the ESWT group (*p* < 0.001), and from 95.6 ± 1.4 mm to 87.2 ± 1.3 mm in the HMT group (*p* < 0.001). The reduction in BMI was also more pronounced in obese participants than in overweight patients, as BMI significantly decreased by 1.95% (*p* < 0.5) in obese participants from the ESWT group and by 2.01% (*p* < 0.01) in obese participants with from the HMT group. HMT was also effective in reducing body fat in obese participants, with a 1.7% decrease (*p* < 0.01). Abdominal obesity significantly decreased by 2.40% (*p* < 0.01) in overweight participants in the HMT group, and by 1.15% (*p* < 0.01) in obese participants in the ESWT group.

### 5.2 Serum lipids

Table 2 shows changes in participants’ blood lipids during the study. No statistically significant difference in total cholesterol was found for any of the subgroups of participants after 12 sessions of ESWT and HMT. However, triglycerides showed a decreasing tendency in overweight participants in the ESWT group, declining from 94.6 ± 14.3 to 70.1 ± 10.0 (*p* = 0.073), and a significant decrease among obese participants in the ESWT group, dropping from 116.1 ± 9.0 to 88.7 ± 9 (*p* < 0.01). There were no significant changes in HDL or cortisol levels in any of the groups, and a decreasing tendency in obese participants from the ESWT group (*p* = 0.086).

**Table 2:** Blood lipids changes in participants during the trial.

		Baseline			12 weeks			Difference
Total cholesterol	Overweight + HMT (n = 7)	203.1	±	9.1	210.9	±	4.5	7.71
	Overweight + ESWT (n = 8)	203.6	±	12.5	201.4	±	13.3	−2.25
	Obese + HMT (n = 13)	193.2	±	11.9	188.0	±	10.9	−5.15
	Obese + ESWT (n = 15)	213.3	±	8.1	204.2	±	8.1	−9.13
Triglyceride	Overweight + HMT (n = 7)	103.7	±	15.3	114.1	±	16.1	10.43†
	Overweight + ESWT (n = 8)	94.6	±	14.3	70.1	±	10.0	−24.50
	Obese + HMT (n = 13)	125.1	±	20.8	116.9	±	20.2	−8.15
	Obese + ESWT (n = 15)	116.1	±	9.0	88.7	±	9.7**	−27.47
HDL-cholesterol	Overweight + HMT (n = 7)	42.9	±	4.0	42.0	±	2.8	−0.86†
	Overweight + ESWT (n = 8)	54.8	±	3.5	53.8	±	3.7	−1.00
	Obese + HMT (n = 13)	45.1	±	2.3	43.8	±	2.1	−1.31
	Obese + ESWT (n = 15)	48.1	±	2.5	47.7	±	2.4	−0.40
LDL-cholesterol	Overweight + HMT (n = 7)	131.0	±	7.8	133.6	±	4.3	2.57
	Overweight + ESWT (n = 8)	118.3	±	10.0	118.5	±	11.7	0.25
	Obese + HMT (n = 13)	115.5	±	10.5	110.4	±	10.4	−5.08
	Obese + ESWT (n = 15)	134.9	±	8.2	126.5	±	7.6	−8.40
Cortisol	Overweight + HMT (n = 7)	9.22	±	1.12	9.86	±	1.28	0.65
	Overweight + ESWT (n = 8)	8.91	±	1.06	8.37	±	1.11	−0.54
	Obese + HMT (n = 13)	9.40	±	0.91	8.44	±	0.79	−0.95
	Obese + ESWT (n = 15)	8.48	±	0.93	9.58	±	0.85	1.11

*p < 0.05, **p < 0.01, ***p < 0.001 compared with baseline.

†p < 0.05 compared with overweight + ESWT.

## 6 Discussion

Although obesity management in Asia often includes ESWT and HMT, clinical research on the effects of these approaches has been lacking. This clinical study investigated the impacts of a six-week (12-session) abdominal treatment with ESWT and HMT on Korean women's serum lipids and body composition, based on their overweight and obesity status.

The study results showed that ESWT and HMT were more effective in obese women than overweight women with regards to changes in serum lipids and body composition. For obese women, changes in body weight, waist circumference, and BMI were statistically significant in both the ESWT and the HMT groups; in particular, obese women in the ESWT group showed a significant reduction in all measurement domains—weight, waist circumference, BMI, body fat, and abdominal obesity percentage. This finding of a superior improvement in weight loss with abdominal management in obese women than overweight women has significant implications because weight loss can reduce the risk of CVD by improving dyslipidemia [22].

Moreover, the finding that HMT reduced body weight and waist circumference is consistent with a study that found a decrease in body weight, abdominal circumference, and appetite with aromatherapy massage in middle-aged women with abdominal obesity [23]. Growing evidence enables us to understand the key role adipose tissue plays in regulating the physiopathological mechanisms and related comorbidities of obesity. Obesity is a major cause of morbidity and mortality, and of various diseases, such as diabetes mellitus, metabolic syndrome, cardiovascular diseases, fatty liver, and cancer [24]. A previous study demonstrated that a reduction in BMI lowered the risk of coronary heart disease in highly obese male and female young adults [25]; the effects of both ESWT and HMT on reducing BMI are applicable to obese women. WHR is the best discriminator for dyslipidemia [26]. It is therefore particularly meaningful that waist size is the only variable showing a significant reduction in both the ESWT and HMT groups. While a previous study on the use of noninvasive ultrasound for intensive therapy demonstrated a reduction in fat thickness in the abdomen, thigh, or flank [27], in the present study, a significant decrease in body fat and abdominal obesity percentage was observed only in the ESWT group.

In contrast to a previous finding of reduced triglycerides (weighted mean difference [WMD] 0.2 mmol/L, 95% confidence interval [CI] −0.3 to −0.1) with a combined treatment of diet and exercise [28], no significant difference was observed in any of the measurement domains for serum lipids in the HMT group, and the only significant reduction observed for triglycerides was in the ESWT group with obese (*p* < 0.01). ESWT generated these results for triglycerides in overweight women and LDL in obese women, indicating the need for follow-up. The substantial reduction in triglycerides among patients managed with ESWT in the present study is consistent with the finding of significant improvements in serum lipids as metabolic parameters from combined ultrasonic cavitation and electrolipolysis [29]. A previous study on the effects of massage and high-frequency therapy on muscle relaxation in middle-aged women found no significant changes in triglyceride, HDL, or LDL levels [30]. Similarly, no significant findings were observed for total cholesterol, HDL, or cortisol levels in any of the subgroups.

ESWT and HMT, which usually are used in Korea and other parts of Asia, are effective management regimens for obese and overweight people that can improve serum lipids and abdominal obesity through positive body measurement changes. However, due to a lack of previous research on ESWT for obesity management, discussion of our study findings was limited.

Moreover, in this study, HMT was treated as a comparative experimental group for ESWT, but there were no intervention control groups. Therefore, the effect of ESWT and HMT should be confirmed by the results before and after treatment. There was a limitation that the comparison of subjects was limited. During the study, participants were unable to control their dietary intake and physical activity and the short study period and the small sample size made it challenging to formulate inferences about common results. Therefore, further research on various related topics and perspectives, including the effects of combined management regimens with the device and hand massage, on a sample with greater age diversity is needed in the future.

## 7 Conclusions

This research has particular significance as the first comparative analysis of the effects of ESWT and HMT on serum lipids and body composition in obese and overweight people. In conclusion, ESWT and HMT could be helpful for the abdominal management of overweight status and obesity. Moreover, ESWT improves body measurements and lowers triglycerides better than HMT—with a more significant effect in individuals with obese than overweight, suggesting an improvement in CVD and a superior impact on health.

This study confirmed that ESWT and HMT can be a management plan for abdominal obesity, a risk factor for chronic diseases. In addition, the results of this study show that HMT for more than 6 weeks (twice a week) was effective for overweight and ESWT was effective for the obese people. The significance of this study suggests a guideline for customized abdominal management according to the degree of obesity applicable to clinicians in the clinical field.

## Author contributions

Soo Yeon Oh : Conceptualiation, Methodology, Software

Kyung jin Lee : Data curation, Writing-Originial draft, Preparation, Writing-Reviewing and Editing

Jin Ik Park : Visualization, Investigation

## Ethical approval

Youngsan University Institutional Review Board Protocol NO: YSUIRB-201510-BR-003-02.

## Research Registration Unique Identifying Number (UIN)

1. Name of the registry: Clinical Research information Service, CRIS.

2. Unique Identifying number or registration ID: KCT0004999.

3. Hyperlink to your specific registration (must be publicly accessible and will be checked): https://cris.nih.go.kr/cris/search/search_result_st01_kren.jsp?seq=16308&sLeft=2&ltype=my&rtype=w International Clinical Trials Registry Platform, http://www.who.int/ictrp.

## Guarantor

Soo Yeon Oh.

At the time this study was conducted, no notice of data sharing was given to the participants. So I decided not to share data.

## Funding

This research did not receive any specific grant from funding agencies in the public, commercial, or not-for profit sectors.

## Provenance and peer review

Not commissioned, externally peer reviewed.

## Declaration of competing interest

None.
